# A Newly Developed Indicator of Overeating Saturated Fat Based on Serum Fatty Acids and Amino Acids and Its Association With Incidence of Type 2 Diabetes: Evidence From Two Randomized Controlled Feeding Trials and a Prospective Study

**DOI:** 10.3389/fnut.2022.897375

**Published:** 2022-06-14

**Authors:** Wei Wei, Tianqi Zi, Ruiming Yang, Jiaxu Xu, Yunyan Chen, XiTao Jiang, Xia Chu, Xue Yang, Wenbo Jiang

**Affiliations:** ^1^Department of Nutrition and Food Hygiene, National Key Discipline, School of Public Health, Harbin Medical University, Harbin, China; ^2^Key Laboratory of Cardiovascular Research, Department of Pharmacology, College of Pharmacy, Ministry of Education, Harbin Medical University, Harbin, China; ^3^College of Engineering, IT and Environment, Charles Darwin University, Darwin, NT, Australia; ^4^Department of Cardiology, The First Affiliated Hospital of Harbin Medical University, Harbin, China

**Keywords:** type 2 diabetes, biomarker, serum fatty acids, saturated fat intake, serum amino acid

## Abstract

**Objective:**

Hyper-caloric intake of saturated fatty acids (SFAs) is common in modern societies, probably contributing to the epidemic of type 2 diabetes mellitus (T2DM). This study conducted two randomized controlled trials (RCTs) for developing a new indicator that can assess the nutritional status and examined its association with incidence of T2DM.

**Methods:**

In RCT 1, healthy participants were randomly assigned into three groups, namely, control group (*n* = 40), overfeeding group 1 (100 g butter per day, *n* = 37), and overfeeding group 2 (120 g butter per day, *n* = 37). In RCT 2, healthy subjects were randomly assigned into two groups, namely, control group (*n* = 52) and high-fat group (300-extra kcal/day from diet that was designed by high-fat diet, *n* = 58). In the prospective cohort, 4,057 participants aged 20–74 years were enrolled and followed up over 5.3 years. Serum profiles of fatty acids and amino acids were measured.

**Results:**

In RCT 1, serum fatty acids, including C14:0 and C18:0, increased, whereas C18:2, C20:4, C22:5, and C22:6 decreased; serum amino acids, including tyrosine, alanine, and aminobutyric acid, increased, whereas histidine and glycine decreased (*p* < 0.05). Among these serum fatty acids and amino acids, changes in C14:0, C20:4, tyrosine, histidine, and glycine were also observed in RCT 2. An indicator was developed based on the five fatty acids and amino acids, namely, C14:0 × tyrosine × 1,000/[C20:4 × (glycine + histidine)], and it significantly identified participants in the intervention group with area under the curve (AUC) (95% CI) being 0.85 (0.77–0.92). The indicator was significantly associated with incidence of T2DM in the prospective cohort with HRs (95% CIs) from bottom quartile to top quartile being 1,1.21 (0.82–1.77), 1.60 (1.12–2.30), 2.04 (1.42–2.94).

**Conclusion:**

The newly developed indicator in RCTs can be used in assessing the nutritional status of hypercaloric intake of SFA and predicting the development of T2DM.

## Introduction

The rapid increase in the prevalence of diabetes mellitus and its complications have been major global health threats. Globally, it has been estimated that 1 of 11 adults currently have diabetes mellitus, and 90% of them have type 2 diabetes mellitus (T2DM) (1). A healthy diet has been demonstrated to play a critical role in the prevention of T2DM. The association between saturated fatty acids (SFAs) and T2DM has been one of the most vexed issues in this field (2, 3). Although the short-term randomized controlled trials (RCTs) have shown that overeating SFA promotes hepatic fat storage and insulin resistance (IR) (4, 5), evidence from a meta-analysis of long-term epidemiological studies frequently shows that dietary SFA is not associated with the incidence of T2DM (6, 7).

Dietary fat has been widely considered as one of the most difficult dietary components to assess through traditional nutritional methods (8). This may be the reason for the inconsistencies in the findings from the short-term RCTs and long-term epidemiological studies. Therefore, it is important to identify biomarkers that can assess the nutritional status of hypercaloric intake of dietary SFA objectively. Previous studies frequently focused on evaluating the utility of fatty acid profiles in serum as biomarkers of dietary SFA. However, serum SFA did not correlate well with dietary intake of SFA (9). Moreover, the total circulating fatty acids and other types of fatty acids, including monounsaturated fatty acids (MUFAs) and polyunsaturated fatty acids (PUFAs), also influence the circulating levels of SFA. Experimental evidence from human studies shows that it is energy intake that is a key mediator of serum fatty acids, rather than dietary fatty acid intake *per se*. The serum profiles of fatty acids may reflect the excessive intake of energy. In addition, recent studies have reported the impact of a high-fat diet on the catabolism of the amino acids (10–12), suggesting that serum profiles of amino acids may be related to the intake of SFA. However, whether and how serum profiles of amino acids would change during feeding of SFA have not been reported in previous RCTs. Examining the serum profiles of amino acids across different interventions of SFA feeding may aid in identifying some new biomarkers that are sensitive to the dietary intake of SFA, and it may be more accurate to evaluate the nutritional status of the hypercaloric intake of SFA by combination of serum fatty acids and amino acids.

In this study, two RCTs by feeding a hypercaloric diet rich in SFA with different intervention doses and sources were performed for identifying fatty acids and amino acids that could be used to assess this nutritional status. We aimed to develop a new indicator based on serum fatty acids and amino acids and examine the association between this newly developed indicator and the incidence of T2DM in a prospective cohort.

## Materials and Methods

### Participants in the Two Randomized Controlled Trials

Participants in the two RCTs were recruited from voluntary and healthy students in Harbin Medical University from March to August 2019. The RCTs were registered at www.chictr.org as ChiCTR-1900021716 and ChiCTR-1900024931, respectively. Exclusion criteria included individuals who were older than 30 years, alcohol drinkers or smokers, had dysfunction of the liver, kidney, or digestive system, dyslipidemia, hypertension or taking medications, took nutritional supplements in the past 2 months, lost weight by restricting diet in the past 6 months, and had vigorous physical activity more than 5 h/week. Based on these exclusion criteria, 23 and 31 individuals were excluded in RCT 1 and RCT 2, respectively.

The participants in the two RCTs were provided with standard meals for 3 days, and then they were randomly assigned by a biostatistician to the intervention and control groups. In RCT 1, healthy participants were randomly assigned into three groups, namely, control group (2,150 kcal/day, 25% fat and 5% SFA, *n* = 40), overfeeding group 1 (100 g butter per day, *n* = 37), and overfeeding group 2 (120 g butter per day, *n* = 37). The same meals during the intervention in the three groups were provided, which is shown in Supplementary Table 1. The excess calories ranging from 100 to 120 g of butter were added to the meals, which contained 76% energy provided by SFA, 20% energy provided by MUFAs, and 4% energy provided by PUFAs), and the energy and macronutrients for the three groups are presented in Supplementary Table 2. In RCT 2, healthy subjects were randomly assigned into two groups, namely, the control group (2,250 kcal/day, 20% fat and 5% SFA, *n* = 52) and high-fat group (300 kcal/day extra energy, *n* = 58). The high-fat diet contained 35% energy provided by total fat and 15% energy provided by SFA, and the detailed information in terms of food groups and macronutrients is presented in Supplementary Tables 3, 4. The percentage of energy provided by protein was similar between the two groups. All the meals in the two RCTs were finished in the student canteen under the supervision of the study dietitian with the intervention periods in the two RCTs lasting for 7 days. Before the two RCTs, the food frequency questionnaire (FFQ) surveys were conducted to collect the dietary information of each participant for evaluating their habitus of dietary intake, and used as the reference for setting the energy and macronutrients provided in the control group. The cooking menu was different between the two RCTs because of the different sources of SFA, making the energy provided in the control group of RCT 2 a bit higher than that of RCT 1 for controlling the overall carbohydrate and protein constant. Moreover, the degree of overnutrition in RCT 1 was greater than RCT 2 because RCT 1 aimed to capture more changes in the profiles of fatty acids and amino acids, and RCT 2 aimed to examine which fatty acids and amino acids could be still significantly changed if the overnutrition was relatively moderate. The overlapped serum fatty acids and amino acids between the two RCTs were used to develop the indicator for the hypercaloric intake of SFA.

### Participants in the Prospective Cohort

The data of the Harbin Cohort Study on Diet, Nutrition and Chronic Non-communicable Disease (HDNNCDS) were used, which was launched in 2010, registered at www.chictr.org as ChiCTR-ECH-12002721. A total of 9,734 participants were recruited in the baseline survey of HDNNCDS. During 2015–2016, 8,913 participants completed the first in-person follow-up survey (13). Among the 8,913 participants, 4,958 participants’ blood samples were randomly selected to measure the serum profiles of fatty acids and amino acids. After exclusion of the participants who had T2DM at baseline, a total of 4,057 subjects aged 20–74 years, including 1,352 men and 2,705 women, were enrolled in both the 2010 baseline survey and the 2016 follow-up survey, with a follow-up period of 5.3 years on average.

The study designs of the two RCTs and the prospective study were approved by the Ethics Committee of Harbin Medical University. The nature and potential risks of the study were explained to volunteers before obtaining written informed consent. The investigations were conducted in accordance with the Declaration of Helsinki. The methods in this study were in accordance with the approved guidelines.

### Data Collection in a Prospective Cohort

In-person interviews were administered by trained personnel using a structured questionnaire to collect information on demographic characteristics, dietary habits, lifestyles, physical conditions, and anthropometric characteristics. Current smokers were defined as those who smoked at least 100 cigarettes in a lifetime or smoked every day or currently smoked some days. Current drinkers were defined as those who consumed ≥1 alcohol every month in 12 months before the survey. Regular exercise was defined as any kind of recreational or sports physical activity other than walking for work or life performed at least 30 min for ≥3 days/week. Family history of diabetes was defined as diabetes in first- or second-degree relatives.

### Anthropometric and Biochemical Measurements

The measurement of height, weight, and waist circumferences (WCs) were conducted by well-trained professionals with participants wearing light, thin clothing, and no shoes. Bodyweight and height were measured to the nearest 0.1 kg and 0.1 cm, and body mass index (BMI) was calculated as weight (kg) divided by the square of the height in meters (m^2^). Fat mass (FM) was measured by using the electric impedance method with a body FM analyzer (ACCUNIQ IOI-353, JAWON Medical Corporation, Korea). The blood samples were collected by venipuncture after a 12-h fast. Plasma and serum were centrifuged from the blood samples and immediately stored at –80°C for further analysis. Fasting serum (FS) glucose, triglyceride (TG), total cholesterol (TC), high-density lipoprotein cholesterol (HDL-C), and low-density lipoprotein cholesterol (LDL-C) were determined by an automatic analyzer (Hitachi 7100, Tokyo, Japan). Serum insulin was measured by Chemiluminescence Immune Analyzer (TOSOH AIA2000, Japan). Glycosylated hemoglobin (HbA1c) was determined by high-performance liquid chromatography (BIO-RAD VARIANT 2, United States). The homeostasis assessment model for IR (HOMA-IR) updated by the University of Oxford in 2004 was used to estimate IR (14). Serum preparation for fatty acids and amino acids quantitation was carried out as previously described (15, 16). Targeted analysis of serum fatty acids was performed by the TRACE GC/Polaris-Q MS system (Thermo Finnigan, Austin, TX, United States). Targeted analysis of serum amino acids was performed by a Waters ACQUITY Ultra performance liquid chromatography (UPLC) system (Waters Corporation, Milford, MA, United States) coupled to a Waters Xevo TQD mass spectrometer (MS) (Waters Corporation, Manchester, United Kingdom). Quality control was conducted using reference standards that were run concurrently with study samples to verify batch-to-batch variation. Absolute concentrations of fatty acids and amino acids were measured with mean interassay coefficients of variation 5.9 and 6.3%.

### Ascertainment of Normal Glycemia, Prediabetes, Insulin Resistance, and Type 2 Diabetes Mellitus in a Prospective Cohort

Normal glycemia was defined as baseline FS glucose <5.5 mmol/L and 2-h glucose <7.8 mmol/L. Prediabetes was defined as baseline FS glucose >5.5 mmol/L, and/or 2-h glucose >7.8 mmol/L. IR was defined as the top quartiles of baseline HOMA-IR. T2DM was defined as self-reports of a history of T2DM diagnosis, FS glucose ≥7.0 mmol/L, 2-h glucose ≥11.1 mmol/L, and/or receiving treatment for T2DM.

### Statistical Analysis

The sample size in RCT 1 was determined based on changes in HOMA-IR (as the primary outcome). At a 5% significance level with 80% statistical power, a sample size of 36 per group was required to detect a 0.60 difference in HOMA-IR with an SD of 0.9. The sample size in RCT 2 was determined based on the area under the curve (AUC) for discriminating the intervention group. At a 5% significance level with 80% statistical power, a sample size of 52 per group was required to detect an AUC value of 0.65. Before analyses, the Kolmogorov–Smirnov test was used to test the normality of distributions, and variables were log-transformed when necessary. The baseline characteristics in the two RCTs and the prospective cohort were compared by Student’s *t*-test for continuous variables and the Chi-square test for categorical variables. Among the two RCTs, the differences (△) between the baseline values and end-point values (end-point value – baseline value) for each participant in terms of anthropometric and biochemical indicators were calculated, and the differences in the △indicators between the control group and intervention group were compared using Student’s *t*-test or one-way ANOVA. Meanwhile, the △fatty acids or △amino acids for each participant were also calculated, which were standardized by zero-mean normalization (*Z*-score) to eliminate the influence of the order of magnitude for different types of fatty acids and amino acids. After the indicator was developed, it was categorized into quartiles in the prospective cohort; the lowest quartile was used as the reference. Cox proportional hazards regression analyses were performed to examine the association between the quartiles of the indicator and incident T2DM in the total sample and subgroups of baseline normal glycemia, prediabetes, IR, men, and women. Three Cox models were conducted with adjustment for a series of covariates. In model 1, age, sex, BMI, WC, weight gain, smoke use, alcohol use, education level, family history of diabetes, energy intake, regular exercise habits, physical activity levels, and hypertension were included, and model 2 additionally adjusted for TG, HDL-C, TC, FS glucose, 2-h glucose, and HOMA-IR based on model 1. Furthermore, model 3 additionally adjusted for serum leucine, isoleucine, and valine based on model 2.

## Results

### Baseline Characteristics of the Two Randomized Controlled Trials

In RCT 1, of the 143 participants assessed for eligibility, 120 participants were randomized, 3 dropped in the group with feeding 100 g butter and 3 dropped in the group with feeding 120 g butter during the study, and 114 participants completed the study. In RCT 2, of the 151 participants assessed for eligibility, 120 were randomized and 10 participants dropped during the intervention, leaving 110 participants in the study. The age, percentage of sex, and dietary intakes at baseline were similar in the two RCTs (Supplementary Table 5). After finishing the intervention, the energy, total fat intake, and saturated fat intake were significantly greater in the two RCTs (Supplementary Table 6).

### Changes in the Body Composition and Biochemical Indicators in the Two Randomized Controlled Trials

The baseline body composition and biochemical indicators were similar across different groups of the two RCTs (Table 1). The mean absolute change of FS glucose in the intervention group of feeding 120 g of butter was significantly greater than that in the control group in RCT 1 (0.14 ± 0.37 vs. 0.33 ± 0.27; *p* = 0.039). Both FS insulin and HOMA-IR were significantly increased in the intervention groups compared with the control groups in the two RCTs (FS insulin: 0.38 ± 3.65 vs. 2.10 ± 4.31 vs. 2.56 ± 4.65, *p* < 0.05 in RCT 1; −0.72 ± 3.69 vs. 1.23 ± 3.63, *p* < 0.01 in RCT 2; HOMA-IR: 0.13 ± 0.79 vs. 0.50 ± 0.93 vs. 0.63 ± 0.97, *p* < 0.01 in RCT 1; −0.15 ± 0.81 vs. 0.29 ± 0.68, *p* < 0.01 in RCT 2).

**TABLE 1:** The anthropometric and biochemical indicators at baseline and mean absolute change after 1 week in the two RCTs.

	RCT 1						RCT 2			
	Control (*N* = 40)		Overfeeding 100 g butter (*N* = 37)		Overfeeding 120 g butter (*N* = 37)		Control (*N* = 52)		HF-diet (35%) (*N* = 58)	
					
	Baseline	Mean absolute change	Baseline	Mean absolute change	Baseline	Mean absolute change	Baseline	Mean absolute change	Baseline	Mean absolute change
BMI (kg/m^2^)	20.6 (2.5)	0.08 (0.31)	20.6 (2.6)	0.23 (0.58)	20.8 (2.7)	0.27 (0.37)	20.9 (2.3)	−0.08 (0.48)	20.8 (1.8)	−0.02 (0.72)
WC (cm)	69.2 (8.8)	1.94 (4.58)	67.5 (9.4)	2.31 (6.31)	70.5 (9.6)	1.68 (4.71)	73.3 (6.6)	−0.68 (2.76)	73.4 (8.6)	−1.30 (3.60)
BF (%)	21.1 (6.0)	−0.10 (2.48)	21.0 (5.7)	0.21 (1.75)	21.8 (5.5)	0.74 (2.35)	19.9 (6.4)	−0.03 (2.09)	19.3 (5.8)	0.31 (2.37)
Weight (kg)	56.5 (9.1)	−0.03 (0.66)	55.7 (9.7)	0.53 (1.28)*	57.9 (11.6)	0.72 (0.70)*	59.6 (8.4)	−1.05 (1.32)	60.0 (9.2)	0.16 (1.01)**
FPG (mmol/L)	4.17 (0.39)	0.14 (0.37)	4.26 (0.23)	0.21 (0.33)	4.18 (0.34)	0.33 (0.27)*	4.10 (0.68)	0.07 (0.71)	4.09 (0.38)	0.20 (0.33)
FINS (pmol/ml)	8.2 (4.0)	0.38 (3.65)	8.9 (4.4)	2.10 (4.31)*	8.5 (3.9)	2.56 (4.96)*	6.2 (3.9)	−0.72 (3.69)	6.1 (3.2)	1.23 (3.63)**
HOMA-IR	1.53 (0.79)	0.13 (0.79)	1.71 (0.87)	0.50 (0.93)**	1.59 (0.74)	0.63 (0.97)**	1.52 (0.91)	−0.15 (0.81)	1.39 (0.73)	0.29 (0.68)**
ALT (U/L)	9.1 (5.5)	2.5 (5.0)	8.8 (3.8)	13.1 (16.2)**	9.5 (5.4)	10.8 (13.2)**	17.2 (11.7)	0.42 (3.1)	19.4 (17.0)	0.21 (13.0)
AST (U/L)	18.8 (3.8)	−1.3 (2.7)	19.2 (3.1)	4.4 (8.9)**	19.8 (3.9)	4.5 (12.4)**	17.9 (4.4)	−0.35 (3.6)	18.5 (6.2)	−0.31 (4.2)
UA (μmol/L)	287.9 (59.1)	−26.9 (31.6)	293.9 (64.2)	−27.6 (28.3)	295.0 (46.2)	−24.9 (27.3)	324.2 (73.0)	6.4 (27.0)	343.8 (95.5)	3.2 (33.8)
TC (mmol/L)	3.90 (0.52)	−0.50 (0.29)	3.95 (0.61)	0.10 (0.30)**	3.99 (0.53)	0.10 (0.30)**	3.96 (0.67)	−0.02 (0.36)	4.11 (0.69)	0.35 (0.43)**
TG (mmol/L)	0.70 (0.21)	−0.07 (1.52)	0.73 (0.32)	−0.10 (0.20)	0.71 (0.18)	−0.15 (0.13)	0.84 (0.40)	0.12 (0.31)	0.98 (0.45)	−0.02 (0.46)
HDL-C (mmol/L)	1.47 (0.31)	−0.04 (0.25)	1.44 (0.26)	0.14 (0.14)**	1.46 (0.29)	0.15 (0.14)**	1.32 (0.25)	−0.07 (0.13)	1.30 (0.30)	0.09 (0.11)**
LDL-C (mmol/L)	2.35 (0.48)	−0.37 (0.29)	2.51 (0.61)	0.03 (0.26)**	2.53 (0.47)	0.04 (0.32)**	1.92 (0.44)	0.003 (0.21)	2.05 (0.52)	0.17 (0.29)**

*Data are mean (SD); BMI, body mass index; WC, waist circumference; BF, percentage of body fat; FPG, fasting glucose; HOMA-IR, homeostasis model assessment for insulin resistance; UA, uric acid; TC, total cholesterol; TG, triglycerides; HDL-C, high-density lipoprotein cholesterol; LDL-C, low-density lipoprotein cholesterol.*

*p < 0.05 for the difference being different from 0.

**p < 0.01 for the difference being different from 0.

### Changes in the Serum Profiles of Fatty Acids and Amino Acids in the Two Randomized Controlled Trials

**FIGURE 1:**
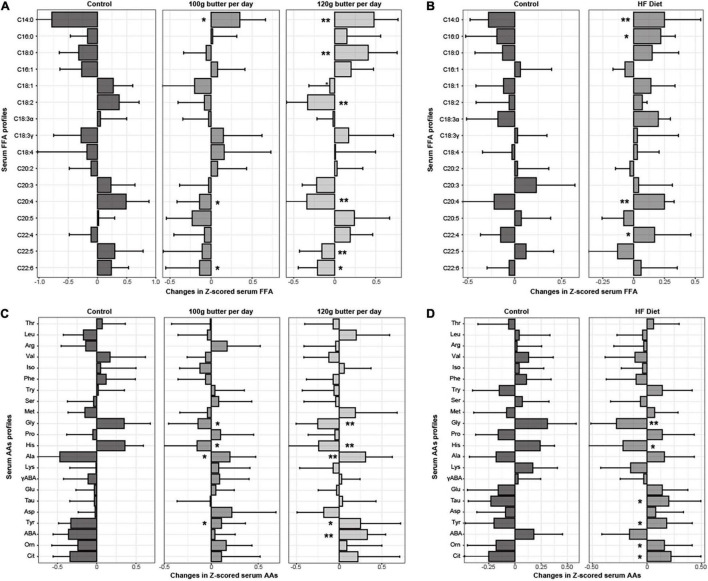
Changes in *Z*-scored profiles of serum fatty acids and amino acids in the two RCTs. *p < 0.05 for the differences in the *Z*-scored serum fatty acids and amino acids between the intervention group and control group. **p < 0.01 for the differences in the *Z*-scored serum fatty acids and amino acids between the intervention group and control group. **(A)** Changes in the *Z*-scored profiles of fatty acids in the RCT1. **(B)** Changes in the *Z*-scored profiles of fatty acids in the RCT2. **(C)** Changes in the *Z*-scored profiles of amino acids in the RCT1. **(D)** Changes in the *Z*-scored profiles of amino acids in the RCT2.

In RCT 1, the baseline serum profiles of fatty acids and amino acids were similar across the three groups (Supplementary Table 7). The significant differences for C14:0, C16:0, C18:0, C18:2, C20:3, C20:4, C22:5, and C22:6 across the three groups after the intervention were observed (Supplementary Table 8). Similarly, △C14:0 and △C18:0 significantly increased in the intervention group with *p*-values being 2.94e^–9^ and 0.010, and the △C18:2, △C20:4, △C22:5, and △C22:6 significantly decreased in the intervention group with *p*-values being 0.010, 0.001, 2.45e^–3^, and 0.044, respectively (Figure 1A). Meanwhile, the significant differences for the γ-aminobutyric acid, tyrosine, ornithine, histidine, glycine, citrulline, alanine, and L-aminobutyric acid across the three groups after the intervention were observed (Supplementary Table 8). Also, △tyrosine, △alanine, and △aminobutyric acid significantly increased in the intervention group with *p*-values being 0.027, 0.001, and 0.009, respectively, and the △glycine and △histidine significantly decreased in the intervention group with *p*-values being 0.017 and 0.014 (Figure 1B).

In RCT 2, the baseline serum profiles of fatty acids and amino acids were similar between the two groups (Supplementary Table 7). The significant differences for the C14:0, C18:2, C18:3α, and C20:4 between the two groups after the intervention were observed (Supplementary Table 8), and △C14:0 and △C16:0 significantly increased in the intervention group with *p*-values being 0.005 and 0.024, and the △C18:3α and △C20:4, significantly decreased in the intervention group with *p*-values being 0.034, and 0.010, respectively (Figure 1C). Meanwhile, the significant differences between the tyrosine, histidine, glycine, and citrulline between the two groups after the intervention were observed (Supplementary Table 8), and △tyrosine, △taurine, and △citrulline acid significantly increased in the intervention group with *p*-values being 0.044, 0.023, and 0.012, respectively, and the △glycine and △histidine significantly decreased in the intervention group with *p*-values being 0.016 and 0.01 (Figure 1D).

### Assessment of the Ability for Discriminating Participants in Randomized Controlled Trial 2

**FIGURE 2:**
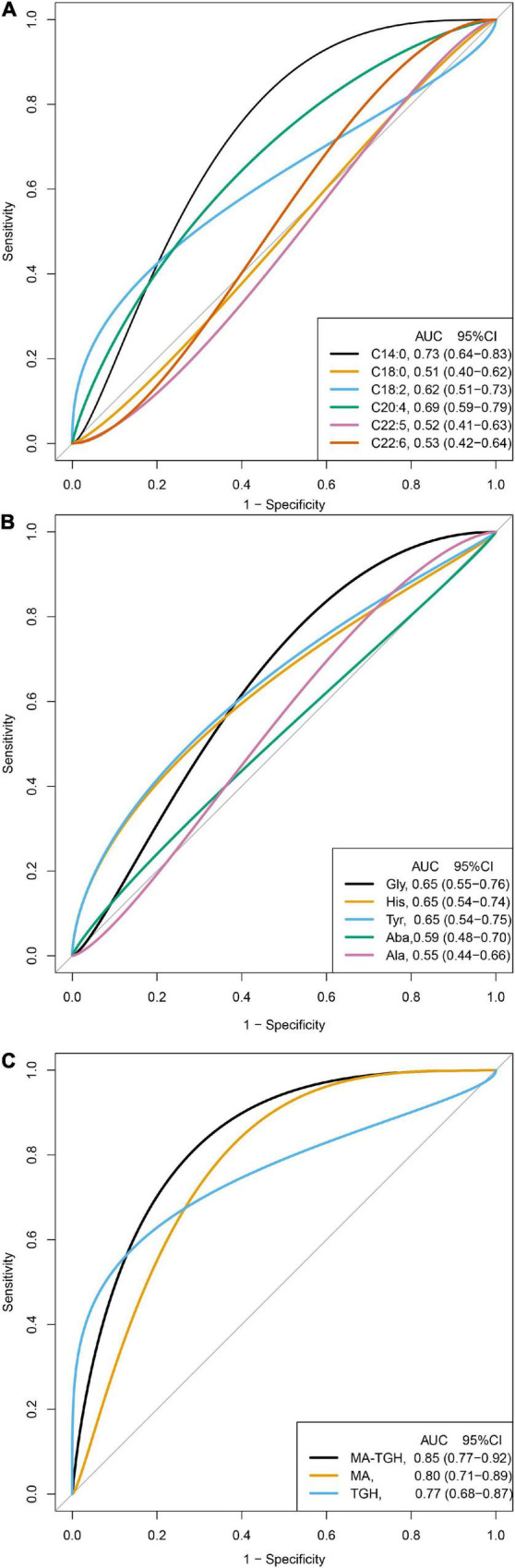
Discrimination ability of the fatty acids, amino acids, and the indicator for identifying participants in the intervention group of RCT 2 (*N* = 110). MA, the ratio of myristic acid (C14:0) to arachidonic acid (20:4); TGH, the ration of tyrosine to the sum of glycine and histidine; MA-TGH, multiplying MA by TGH calculated by C14:0 × tyrosine × 1,000/[C20:4 × (glycine + histidine)]. **(A)** Discrimination ability of the fatty acids in the RCT2. **(B)** Discrimination ability of the amino acids in the RCT2. **(C)** Discrimination ability of combined indicators in the RCT2.

Because the levels of C14:0, C18:0, C18:2, C20:4, C22:5, and C22:6 were significantly different after the intervention among the three groups, and their significant changing values in terms of △ were also observed in the intervention groups of RCT 1, we therefore further examined which of the above fatty acids could be used indiscriminate the participants who were in the intervention group in RCT 2, which is shown in Figure 2A. C14:0, C:18:2, and C20:4 could identify participants in the intervention group of RCT 2 with the AUCs (95% CI) being 0.73 (0.64–0.83), 0.62 (0.51–0.73), and 0.69 (0.59–0.79), whereas the AUCs (95% CIs) for C18:2, C22:5, and C22:6 were 0.51 (0.40–0.62), 0.52 (0.41–0.63), and 0.53 (0.42–0.64), where 95% CIs crossed 0.5, suggesting that the discriminating abilities of these fatty acids were non-significant. Meanwhile, in RCT 2, no significant changes in C18:2 were observed during the intervention; we, therefore, excluded C18:2 as described in Figure 1C and Supplementary Table 5, and calculated the ratio of myristic acid (C14:0) to arachidonic acid (20:4) (MA) based on the direction of changes of the two fatty acids to examine whether the combination of the two fatty acids could elevate the discriminating abilities for identifying the participants with a high-fat diet. The MA could identify the participants more accurately with the AUC (95% CI) being 0.80 (0.71–0.89) (Figure 2C).

Similarly, because the levels of glycine, histidine, tyrosine, alanine, and L-aminobutyric were significantly different after the intervention among the three groups, and their significant changing values in terms of △ were also observed in the intervention groups of RCT 1, we therefore further examined which of the above amino acids could be used to indiscriminate the participants who were in the intervention group in RCT 2, which is shown in Figure 2B. Glycine, histidine, and tyrosine could identify participants in the intervention group of RCT 2 with the AUCs (95% CI) being 0.65 (0.55–0.76), 0.65 (0.54–0.74), and 0.65 (0.54–0.75), whereas the AUCs (95% CIs) for alanine and L-aminobutyric were 0.55 (0.44–0.66) and 0.59 (0.48–0.70), where the 95% CIs crossed 0.5, suggesting that the discriminating abilities of the two amino acids were non-significant. Based on the direction of changes of the three amino acids, the ratio of tyrosine to the sum of glycine and histidine (TGH) was calculated to examine whether the combination of the three amino acids could elevate the discriminating abilities for identifying the participants with a high-fat diet. The TGH could identify the participants more accurately with the AUC (95% CI) being 0.77 (0.68–0.87) (Figure 2C).

Furthermore, we also examined whether the combination of these fatty acids and amino acids could elevate the discriminating abilities, and the indicator was further developed by multiplying MA by TGH (MA-TGH): C14:0 × tyrosine × 1,000/[C20:4 × (glycine + histidine)], and we found that it has more accurate discriminating ability than MA or TGH used alone with AUC (95% CI) being 0.85 (0.77–0.92) (Figure 2C).

### Association of MA-TGH With the Incidence of Type 2 Diabetes Mellitus

In this longitudinal data, age, percentage of men, BMI, WC, the prevalence of hypertension, family history of diabetes, blood lipids, FS glucose, 2-h glucose, HOMA-IR, and serum BCAA at baseline were significantly greater in new cases of T2DM (Supplementary Table 9). MA-TGH was significantly associated with weight gain (*r* = 0.136, *p* < 0.001) (Supplementary Figure 1). The association of MA-TGH with the incidence of T2DM is presented in Table 2. After adjustment for age, sex, BMI, WC, smoke use, alcohol use, education level, family history of diabetes, energy intake, regular exercise habits, physical activity levels, hypertension, TG, HDL-C, TC, FS glucose, 2-h glucose, HOMA-IR, valine, leucine, isoleucine, and weight gain in model 4, MA-TGH was significantly associated with the incidence of T2DM in the total sample with HRs (95% CI) from bottom to the top quartiles being 1, 1.17 (0.82–1.72), 1.70 (1.21–2.39), and 2.09 (1.49–2.92). Meanwhile, among the participants with normal glycemia at baseline, MA-TGH was still associated with the incidence of T2DM with HR (95% CI) from bottom to the top quartiles being 1, 1.49 (0.89–2.50), 1.66 (0.99–2.77), and 2.75 (1.68–4.48) after adjustment for the covariates included in model 3 (Table 2), and sex had no effect on this association (Supplementary Table 10).

**TABLE 2:** HRs and 95% CI for the association between the quartiles of the indicator and incidence of T2DM in the total, normal glycemic, prediabetes, and IR samples.

	Quartile 1	Quartile 2	Quartile 3	Quartile 4	*p*_*for trend*_
**Total-sample**					
Case/*N*	51/1,014	65/1,014	99/1,015	150/1,014	
Model 1	1 (Ref.)	1.21 (0.83–1.76)	1.93 (1.36–2.73)	2.93 (2.11–4.07)	<0.001
Model 2	1 (Ref.)	1.21 (0.83–1.76)	1.60 (1.13–2.28)	2.08 (1.47–2.94)	<0.001
Model 3	1 (Ref.)	1.21 (0.82–1.77)	1.60 (1.12–2.30)	2.04 (1.42–2.94)	<0.001
**Normal glycemic**					
Case/*N*	24/822	37/822	41/822	72/822	
Model 1	1 (Ref.)	1.41 (0.82–2.43)	2.23 (1.34–3.72)	3.07 (1.88–5.01)	<0.001
Model 2	1 (Ref.)	1.35 (0.78–2.31)	2.00 (1.20–3.35)	2.51 (1.51–4.18)	<0.001
Model 3	1 (Ref.)	1.40 (0.80–2.43)	2.10 (1.23–3.59)	2.67 (1.54–4.62)	<0.001
**Pre-diabetes**					
Case/*N*	30/193	42/192	52/191	67/193	
Model 1	1 (Ref.)	1.62 (1.00–2.64)	1.84 (1.15–2.94)	2.64 (1.68–4.14)	<0.001
Model 2	1 (Ref.)	1.75 (1.07–2.84)	1.88 (1.17–3.02)	2.54 (1.57–4.10)	<0.001
Model 3	1 (Ref.)	1.74 (1.06–2.84)	1.83 (1.14–2.96)	2.37 (1.44–3.91)	0.001
**IR**					
Case/*N*	18/253	37/253	48/253	52/253	
Model 1	1 (Ref.)	1.74 (1.00–3.04)	2.50 (1.46–4.28)	2.97 (1.76–5.01)	<0.001
Model 2	1 (Ref.)	1.71 (0.98–2.98)	2.18 (1.27–3.74)	2.48 (1.43–4.30)	0.001
Model 3	1 (Ref.)	1.59 (0.91–2.78)	2.00 (1.15–3.48)	1.95 (1.07–3.54)	0.023

*In model 1, age, sex, BMI, WC, weight gain, smoke use, alcohol use, education level, family history of diabetes, energy intake, regular exercise habits, physical activity levels, and hypertension were included.*

*Model 2 additionally adjusted for TG, HDL-C, TC, FS glucose, 2-h glucose, and HOMA-IR based on model 1.*

*Model 3 additionally adjusted for serum leucine, isoleucine, and valine based on model 2.*

*BMI, body mass index; WC, waist circumference; TG, triglycerides; HDL-C, high-density lipoprotein cholesterol; TC, total cholesterol; FS glucose, fasting serum glucose. Normal glycemia was defined as baseline fasting serum (FS) glucose <5.5 mmol/L and 2-h glucose <7.8 mmol/L. Prediabetes was defined as baseline FS glucose >5.5 mmol/L, and/or 2-h glucose >7.8 mmol/L. IR was defined as the top quartiles of baseline HOMA-IR. Ref., reference.*

## Discussion

In this study, two RCTs with the intervention of hypercaloric intake of SFA from different doses and sources were conducted. In RCT 1, numbers of serum fatty acids and amino acids were significantly varied after overeating SFA. Among these serum fatty acids and amino acids, the significant changes in myristic acid (C14:0), arachidonic acid (C20:4), tyrosine, glycine, and histidine during the intervention were consistently observed in RCT 2, which showed lower discrimination ability for identifying the participants with the hypercaloric intake of SFA. An indicator of hypercaloric intake of SFA was therefore developed based on the above five serum fatty acids and amino acids. This indicator could significantly identify participants with hypercaloric intake of SFA in RCT 2, and it was associated with the development of future risk of T2DM, independent of other risk factors, particularly glucose, IR, and branched-chain amino acids. To our knowledge, this is the first study to establish an indicator based on fatty acids and amino acids, which can assess the nutritional status of hypercaloric intake of SFA, and examine its association with T2DM.

The most important finding of this study is that an indicator of hypercaloric intake of SFA was developed in this study. To achieve this, two RCTs were conducted in this study, with one for developing the indicator and the other for validation. In RCT 1, 100 and 120 g butter was provided as the main source of SFA to the participants. Compared to previous studies that reported the composition of serum FFA, this study examined the absolute changed values of serum FFA. We found that serum myristic acid (C14:0), stearic acid (C18:0), tyrosine, alanine, and L-aminobutyric acid increased, and linoleic acid (C18:2), arachidonic acid (C20:4), EPA (C22:5), DHA (C22:6), histidine, and glycine decreased among the participants in the group of 120 g butter per day, and only myristic acid (C14:0), linoleic acid (C18:2), arachidonic acid (C20:4), and EPA (C22:5) among the fatty acids and tyrosine, histidine, and glycine among the amino acids were significantly varied among the participants in the group of 100 g butter per day. This observation suggested that the number of significant changed fatty acids and amino acids gradually decreased with the stimulation of hypercaloric intake of SFA, and myristic acid (C14:0), arachidonic acid (C20:4), EPA (C22:5), tyrosine, histidine, and glycine probably had dose-response manner in relation to the hypercaloric intake of SFA from butter. These observations were consistent with previous studies, all indicating that serum long-chain SFAs increased, and serum unsaturated fatty acids decreased during hypercaloric intake of SFA (4, 5, 17–19). Also, these observations suggested that hypercaloric intake of SFA could influence the metabolic pathways of amino acids, and these amino acids were probably associated with hypercaloric intake of SFA.

Compared to RCT 1, RCT 2 adopted a more moderate degree of hypercaloric intake of SFA with a high-fat diet as the main source of SFA. We observed that myristic acid (C14:0), palmitic acid (C16:0), tyrosine, taurine, and citrulline acid increased, and α-linolenic acid (C18:3α), arachidonic acid (C20:4), histidine, and glycine were decreased. Although the different varied fatty acids and amino acids were observed, probably because of the different sources of SFA, the significant changes in myristic acid (C14:0), arachidonic acid (C20:4), tyrosine, histidine, and glycine were still observed in RCT 2, suggesting that these fatty acids and amino acids were relatively robust with the different sources of SFA. Furthermore, these fatty acids and amino acids showed relatively weak discrimination abilities for identifying the participants with the hypercaloric intake of SFA. Based on these results, this study intended to examine whether their combination could elevate the accuracy of discrimination ability. Therefore, MA-TGH, as a new indicator, was developed, and it could identify the participants who were in the group of hypercaloric intake of SFA with AUC being 0.85, suggesting that this indicator based on serum fatty acids and amino acids may be more accurate than the individual fatty acids or amino acids.

These results could be supported by several studies. Consistent results from previous RCTs have documented the association between SFA intake and serum cholesterol levels (13), which were also observed in the two RCTs of this study, and it has been reported that serum myristic acid has stronger positive cholesterolemic effects than other serum fatty acids (20). Moreover, a previous observational study has shown weak but significant positive and negative association of dietary fat with serum myristic acid (20). Cell and animal studies have indicated that myristic acid has been the first long-chain endogenous SFA in the fatty acid pathways, which can be elongated to palmitic acid by *de novo* lipogenesis (21), and it is more rapidly metabolized from hepatic to circulating than are other long-chain SFA (22). For arachidonic acid, previous feeding trials have shown that a low SFA diet resulted in increased serum arachidonic acid levels (23), and a high intake of SFA prompted arachidonic acid turnover in rats (24). Meanwhile, a previous study has reported that a high-fat diet could decrease tyrosine hydroxylase mRNA expression, resulting in increased plasma tyrosine (25). For glycine, the expression of pathways of glycine catabolism in the liver and urinary excretion of acyl glycines decreased in the high-fat diet (26, 27), and previous intervention studies have shown that plasma glycine decreased after 1 week of a meat diet, in which the intervention sources were mainly used red meat (28). For histidine, an intervention study has reported that histidine increased after feeding lean meat with low fat (29). Moreover, consistent results have documented the anti-inflammatory effect of glycine and histidine in obese people (30–32).

Another important finding of this study is that MA-TGH, as an indicator of hypercaloric intake of SFA, was significantly associated with future risk of T2DM, independent of other traditional risk factors, and this association was still significant in the participants who were normal glycemic. The FFQ is a relatively valid and commonly used instrument to capture diet information in observational studies. It has been found that FFQ frequently underestimates the intake of energy, and this underestimated percentage may be greater among people with obesity or T2DM, although the association between hypercaloric intake and increased risk of T2D has been abundantly documented. The serum fatty acids and amino acids used in MA-TGH have been reported to be associated with T2DM in previous population-based studies. The EPIC study across eight European countries has documented the association between myristic acids and increased risk of T2DM (33), and tyrosine has been consistently reported to be associated with IR, decreased insulin secretion, and the development of T2DM (34, 35). A pooled analysis of 20 prospective cohorts has shown that increased arachidonic acid was associated with reduced risk of T2DM (36), and a recent study based on genetic approaches has demonstrated a protective effect of glycine on T2DM, driven by a glycine-lowering effect of IR (37). It has also been indicated that histidine supplementation could improve IR and suppress inflammation and oxidative stress (38).

These findings of this study have important implications. A recent meta-analysis based on RCT has suggested that replacing dietary SFA with PUFA significantly lowered mortality risks of cardiovascular disease and all-cause (39); therefore, it is important to establish biomarkers that can objectively and sensitively assess the nutritional status of SFA. MA-TGH may aid in identifying individuals with excessive intake of SFA and developing targeted intervention plans for preventing the development of T2DM because the relationships of fatty acids and amino acids in MA-TGH with T2DM have been consistently confirmed in previous studies.

This study has several strengths. First, two RCTs with different doses and sources of SFA intervention were conducted, and the participants in the two RCTs were young medical students with high compliance. Second, the previous study has reported that it is difficult to modify the proportion of circulating fatty acids that are already accumulated in the body, probably leading that serum SFA did not correlate well with dietary intake of SFA (9). The participants in the two RCTs were relatively young, and therefore they may be more sensitive to the hypercaloric intake of SFA. Third, this study examined the association between the biomarker and incidence of T2DM with a relatively large OGTT sample of nutritional and metabolic analyses in this issue.

We also recognized that this study has certain limitations. First, the two RCTs in this study only included young, healthy Asian ethnic, which limits the generalizability of the findings. Compared with other ethnics, absorption and metabolic rates of carbohydrates tend to be higher, whereas that of fat tends to be lower in Asians (40). Future study is still needed to evaluate whether MA-TGH could be used for assessing the nutritional status of overeating SFA in other ethnics. Second, although the percentage of men and women was similar between the intervention group and control group after the randomization allocation, the percentage of women in the two RCTs was relatively high. Third, this study only examined the association between MA-TGH with T2DM in Asian ethnic; however, the associations of fatty acids and amino acids in the indicator with T2DM have been consistently demonstrated across different ethnicities (33–37). We would therefore expect that our observations would hold across other populations. Fourth, this study only examined the profiles of serum fatty acids but in the absence of data on intracellular values in the two RCTs. The serum profiles could be more easily used in clinical and epidemiological studies; however, fatty acids in adipose tissue may be more accurate. Fifth, in the prospective cohort, we could not exclude the possibility that the increased levels of MA-TGH were induced by other factors, such as genetic risk factors. The previous studies have identified a few gene polymorphisms for the fatty acids or amino acids used in MA-TGH (41–44). Therefore, future study is still needed to examine the interaction effects of the hypercaloric intake of SFA, levels of MA-TGH, and gene polymorphism on the development of T2DM to provide more comprehensive evidence for the association between MA-TGH and T2DM. Furthermore, we lacked information on the fatty acids and amino acids at the follow-up. Analyzing the changes in the levels of TAG-MA between baseline and follow-up might provide more compelling evidence for the association between this indicator and the risk of T2DM. Finally, this study did not control the energy intake between the intervention group and control group to be constant. It is unclear whether the indicator could reflect the intake of SFA under the condition that was not overfeeding. Future study with rigorously controlling the energy is warranted to validate the findings in this study.

In conclusion, this study developed and validated an indicator of overeating SFA based on changes in profiles of serum fatty acids and amino acids. The indicator was associated with the development of T2DM. These findings may have implications for the possible modifiable pathways to T2DM.

## Data Availability Statement

The original contributions presented in the study are included in the article/Supplementary Material, further inquiries can be directed to the corresponding authors.

## Ethics Statement

The studies involving human participants were reviewed and approved by the Ethics Committee of Harbin Medical University. The patients/participants provided their written informed consent to participate in this study.

## Author Contributions

XC, XY, and WW conceived the idea. WW and RY drafted the manuscript. WJ, YC, and JX conducted the statistical analyses. WJ and TZ conducted the first feeding trial. XJ, YC, and TZ conducted the second feeding trial. TZ and WW did the amino acid and fatty acids measurements. All authors critically assessed and reviewed the manuscript.

## Conflict of Interest

The authors declare that the research was conducted in the absence of any commercial or financial relationships that could be construed as a potential conflict of interest.

## Publisher’s Note

All claims expressed in this article are solely those of the authors and do not necessarily represent those of their affiliated organizations, or those of the publisher, the editors and the reviewers. Any product that may be evaluated in this article, or claim that may be made by its manufacturer, is not guaranteed or endorsed by the publisher.
