# Supplementary material for: A Newly Developed Indicator of Overeating Saturated Fat Based on Serum Fatty Acids and Amino Acids and Its Association With Incidence of Type 2 Diabetes: Evidence From Two Randomized Controlled Feeding Trials and a Prospective Study
Source: Front Nutr. 2022 Jun 14;9:897375. doi: 10.3389/fnut.2022.897375 (PMC9237542; doi:10.3389/fnut.2022.897375)
Supplement: Supplementary file 1 [file Data_Sheet_1.docx]

| **Day** | **Breakfast** | **Lunch** | **Dinner** | **Snack** |
| --- | --- | --- | --- | --- |
| **Monday** | Egg, 50g Yogurt, 200ml Corn, 130g Sweet potato, 270g | Stewed pork with brown sauce, 50g Pork stew noodles, 150g Sautéed Potato, Green Pepper, Eggplant, 150g Rice, 180g Whole wheat bread, 50g | Hanging ginkgo, 100g Sauteed Kale, 150g Mixed rice, 150g Whole wheat bread, 50g | Apple, 280g Nuts, 25g |
| **Tuesday** | Egg, 50g Milk, 200ml Banana, 150g Oatmeal, 36g | Cola Chicken Wings, 75g Boiled Pork, 150g Rice, 180g Purple potato, 200g Whole wheat bread, 50g | Dried Green Beans, 150g Mixed rice, 150g Sweet potato, 200g Whole wheat bread, 50g | Grape, 300g Nuts, 25g |
| **Wednesday** | Egg, 50g Soy milk, 250ml Fritters, 70g Corn buns, 150g | Sweet potato, 300g Hot and sour shredded potato, 150g Rice, 180g Whole wheat bread, 50g | Meat end eggplant, 150g Stewed pork with brown sauce, 50g Fixed rice, 150g Whole wheat bread, 50g | Orange, 200g Nuts, 25g |
| **Thursday** | Corn flake, 100g Pumpkin porridge, 300g Egg, 50g Beef buns, 100g | Spicy Hot Pot, 200g Rice, 180g Whole wheat bread, 50g | Fish-flavored shredded pork, 150g Whole wheat bread, 50g Corn buns, 150g Casserole Cabbage Vermicelli Soup, 400g | Apple, 160g Nuts, 25g |
| **Friday** | Black rice porridge, 150g Egg, 50g Purple potato, 200g Potato, 200g Corn flake, 100g | Curry beef, 150g Stir-fried vegetables, 150g Rice, 180g Whole wheat bread, 50g | Braised Chicken Thighs, 100g Eggplant with Minced Meat Sauce, 100g Mixed rice, 150g Whole wheat bread, 50g | Pitaya, 100g Nuts, 25g |
| **Saturday** | Vegetable Noodles, 150g Milk, 200ml Purple potato, 100g Egg, 50g Corn flake, 100g | Purple potato, 200g Steamed Egg with Shrimp, 150g Soy potato shreds, 150g Whole wheat bread, 50g | Bake toufu of Japanse style, 150g Sweet potato, 300g Rice, 180g Whole wheat bread, 50g | Banana, 150g Nuts, 25g |
| **Sunday** | Black rice porridge, 200g Pork cabbage buns, 150g Steamed sweet potato,130g Egg, 50g | Boiled sole Fish, 100g Dry-fried shredded pork, 150g Whole wheat bread, 50g | Fried mushrooms, 100g Mixed rice, 150g Whole wheat bread, 50g | Banana, 125g Nuts, 25g |

Supplementary Table 1 the Cooking menu used in the RCT1

Supplementary Table-2 Energy and macronutrients that was provided in the three groups of RCT1

|  | **Control** | **100g Butter** | **120g Butter** |
| --- | --- | --- | --- |
| **Energy (kcal/d)** | 2392.92 | 3280.57 | 3458.17 |
| **Protein (g/d)** | 79.89 | 81.29 | 81.57 |
| **Carbohydrate (g/d)** | 345.34 | 345.34 | 345.34 |
| **Total Fat (g/d)** | 76.89 | 174.89 | 194.49 |
| **SFA (g/d)** | 27.96 | 102.44 | 118.35 |
| **MUFA (g/d)** | 36.72 | 56.32 | 60.24 |
| **PUFA (g/d)** | 18.49 | 22.41 | 23.19 |

Supplementary Table-3 the Cooking menu used in the RCT2

| **Day** | **Group** | **Breakfast** | **Lunch** | **Dinner** | **Snack** |
| --- | --- | --- | --- | --- | --- |
| **Monday** | **Control** | Black rice porridge, 200g Pork cabbage buns, 150g Steamed sweet potato,130g Egg, 50g | Steamed Egg with Shrimp, 150g Pork stew noodles, 150g Sautéed Potato, Green Pepper, Eggplant, 150g Rice, 180g Whole wheat bread, 60g | Hanging ginkgo, 100g Sauteed Kale, 150g Mixed rice, 150g Whole wheat bread, 60g | Apple, 280g Nuts, 25g |
|  | **HF-Diet** | Egg, 50g Yogurt, 200ml Corn, 130g Sweet potato, 270g | Sautéed Potato, Green Pepper, Eggplant, 150g Rice, 180g  Whole wheat bread, 100g  Fried mushrooms, 150g | Hanging ginkgo, 100g Sauteed Kale, 150g Mixed rice, 150g Braised ribs, 150g |  |
| **Tuesday** | **Control** | Egg, 50g Milk, 200ml Banana, 150g Oatmeal, 36g | Cola Chicken Wings, 75g Boiled Pork, 150g Rice, 180g Purple potato, 200g Whole wheat bread, 60g | Dried Green Beans, 150g Mixed rice, 150g Sweet potato, 200g Whole wheat bread, 60g | Grape, 300g Nuts, 25g |
|  | **HF-Diet** | Egg, 50g Milk, 200ml Banana, 150g Oatmeal, 36g | Rice, 180g Purple potato, 200g Blanched Broccoli with Shrimp, 150g Braised elbows, 150g | Dried Green Beans, 150g Fried rice, 150g Sweet and Sour Pork, 150g Eggplant with minced meat, 150g |  |
| **Wednesday** | **Control** | Egg, 50g Soy milk, 250ml Fritters, 70g Corn buns, 150g | Fried Shredded Pork with Garlic Moss, 150g Hot and sour shredded potato, 150g Rice, 180g Whole wheat bread, 60g | Kung Pao chicken, 150g Fried Potatoes with Garlic Moss, 150g Fixed rice, 150g Whole wheat bread, 60g | Orange, 200g Nuts, 25g |
|  | **HF-Diet** | Egg, 50g Soy milk, 250ml Fritters, 70g Corn buns, 150g | Fried Shredded Pork with Garlic Moss, 150g Cantonese sausage, 100g Braised ribs, 150g Rice, 180g | Kung Pao chicken, 150g Fried Potatoes with Garlic Moss, 150g Fixed rice, 150g |  |
| **Thursday** | **Control** | Corn flake, Pumpkin porridge, 300g Egg, 50g Beef buns, 100g | Spicy Hot Pot, 200g Rice, 180g Whole wheat bread, 60g | Fish-flavored shredded pork, 150g Whole wheat bread, 60g Corn buns, 150g Casserole Cabbage Vermicelli Soup, 400g | Apple, 160g Nuts, 25g |
|  | **HF-Diet** | Corn flake, 100g Pumpkin porridge, 300g Egg, 50g Beef buns, 100g | Spicy Hot Pot, 200g Rice, 180g | Corn buns, 150g Casserole Cabbage Vermicelli Soup, 400g Black pepper steak, 150g Braised Pork, 50g |  |
| **Friday** | **Control** | Black rice porridge, 150g Egg, 50g Purple potato, 200g Potato, 200g Corn flake, 100g | Curry beef, 150g Stir-fried vegetables, 150g Rice, 180g Whole wheat bread, 60g | Braised Chicken Thighs, 100g Eggplant with Minced Meat Sauce, 100g Mixed rice, 150g Whole wheat bread, 60g | Pitaya, 100g Nuts, 25g |
|  | **HF-Diet** | Black rice porridge, 150g Egg, 50g Purple potato, 200g Potato, 200g Corn flake, 100g | Curry beef, 150g Stir-fried vegetables, 150g Rice, 180g Dried shredded pork, 150g | Braised Chicken Thighs, 100g Eggplant with Minced Meat Sauce, 100g Mixed rice, 150g Five Spice Braised Chicken Thighs, 100g |  |
| **Saturday** | **Control** | Vegetable Noodles, 150g Milk, 200ml Purple potato, 100g Egg, 50g Corn flake, 100g | Purple potato, 200g Steamed Egg with Shrimp, 150g Soy potato shreds, 150g Whole wheat bread, 60g | Bake toufu of Japanse style, 150g Sweet potato, 300g Rice, 180g Whole wheat bread, 60g | Banana, 150g Nuts, 25g |
|  | **HF-Diet** | Vegetable Noodles, 150g Milk, 200ml Purple potato, 100g Egg, 50g Corn flake, 100g | Purple potato, 200g Braised ribs, 150g Dried shredded pork, 50g | Bake toufu of Japanse style, 150g Rice, 180g Eggplant with Minced Meat Sauce, 150g |  |
| **Sunday** | **Control** | Egg, 50g Yogurt, 200ml Corn, 130g Sweet potato, 270g | Boiled sole Fish, 100g Slippery meat, 150g Whole wheat bread, 60g | Sweet and Sour Pork, 150g Fried mushrooms, 100g Mixed rice, 150g Whole wheat bread, 60g | Banana, 125g Nuts, 25g |
|  | **HF-Diet** | Black rice porridge, 200g Pork cabbage buns, 150g Steamed sweet potato,130g Egg, 50g | Boiled sole Fish, 150g Slippery meat, 150g Fried rice, 150g Braised Pork, 150g | Sweet and Sour Pork, 150g Fried mushrooms, 100g Mixed rice, 150g  Whole wheat bread, 20g |  |

Supplementary Table 4 Energy and macronutrients that was provided in the two groups of RCT2

|  | **Control** | **HF-Diet** |
| --- | --- | --- |
| **Energy (kcal/d)** | 2350.8 | 2621.11 |
| **Protein (g/d)** | 109.2 | 102.0 |
| **Carbohydrate (g/d)** | 343.5 | 311.63 |
| **Total Fat (g/d)** | 60.0 | 107.4 |
| **SFA (g/d)** | 24.4 | 44.8 |
| **MUFA (g/d)** | 33.0 | 55.8 |
| **PUFA (g/d)** | 19.3 | 22.4 |

Supplementary Table-5 The baseline demographic and dietary intakes in the two RCTs

|  | RCT1 | | | | RCT2 | | |
| --- | --- | --- | --- | --- | --- | --- | --- |
|  | Control  (N=40) | 100g butter per day  (N=37) | 120g butter per day  (N=37) | *P* -value | Control  (N=52) | HF-diet  (N=58) | *P*-value |
| Age (years) | 22.9 (1.36) | 22.2 (1.48) | 22.5 (1.47) | 0.157 | 22.1 (1.23) | 22.4 (1.35) | 0.624 |
| Male [N (%)] | 9 (24.3) | 8 (22.8%) | 10 (25.6) | 0.336 | 14 (26.9) | 16 (27.6) | 0.872 |
| Energy (kcal/d) | 2147 (261) | 2133(260) | 2190(285) | 0.856 | 2251 (196) | 2265 (233) | 0.886 |
| Protein (g/d) | 74.3 (6.2) | 74.3 (7.8) | 74.1 (8.2) | 0.984 | 79.1 (7.7) | 79.6 (8.1) | 0.578 |
| Carbohydrate (g/d) | 323.8 (27.0) | 326.8(31.2) | 328.1(30.4) | 0.888 | 334.1 (14.8) | 336.1(16.1) | 0.601 |
| Total Fat (g/d) | 58.4 (9.4) | 56.2 (12.2) | 59.1 (12.9) | 0.615 | 50.7 (4.3) | 51.8 (9.8) | 0.531 |
| SFA (g/d) | 14.1 (1.9) | 13.4 (3.1) | 14.9 (4.2) | 0.523 | 17.7 (3.4) | 18.7 (5.5) | 0.622 |
| C14:0 (g/d) | 0.3 (0.1) | 0.2 (0.1) | 0.4 (0.6) | 0.882 | 0.6 (0.1) | 0.8 (0.3) | 0.702 |
| C15:0 (g/d) | 0.1 (0.1) | 0.1 (0.1) | 0.1 (0.1) | 0.923 | 0.03 (0.01) | 0.03 (0.01) | 0.991 |
| C16:0(g/d) | 10.5 (1.6) | 10.2 (1.6) | 10.9 (1.9) | 0.871 | 11.8 (1.6) | 11.6 (4.7) | 0.756 |
| C17:0 (g/d) | 0.1 (0.1) | 0.1 (0.1) | 0.1 (0.1) | 0.933 | 0.1 (0.1) | 0.1 (0.1) | 0.882 |
| C18:0 (g/d) | 3.1 (0.5) | 2.9 (0.9) | 3.1 (1.9) | 0.972 | 4.7 (0.8) | 4.9 (1.7) | 0.661 |
| C19:0 (g/d) | 0.15 (0.02) | 0.15 (0.02) | 0.15 (0.05) | 0.773 | 0.05 (0.01) | 0.04 (0.02) | 0.281 |
| C20:0 (g/d) | 0.12 (0.02) | 0.11 (0.02) | 0.13 (0.03) | 0.536 | 0.18 (0.03) | 0.18 (0.10) | 0.991 |
| C22:0 (g/d) | 0.05 (0.01) | 0.05 (0.01) | 0.05 (0.01) | 0.992 | 0.02 (0.01) | 0.02 (0.01) | 0.996 |
| MUFA (g/d) | 24.9 (3.3) | 24.1 (4.0) | 25.2 (5.5) | 0.762 | 22.7 (3.2) | 22.9 (6.3) | 0.921 |
| PUFA (g/d) | 13.4 (1.8) | 13.3 (1.0) | 13.5 (1.8) | 0.972 | 13.6 (2.4) | 13.7 (2.1) | 0.736 |

Continuous variables was presented as mean and SD; Categorical variables was presented as numbers and percentage; SFA, saturated fatty acid; MUFA, monounsaturated fatty acids; PUFA, polyunsaturated fatty acids;

One-way ANOVA and t-test was used to probe the differences across groups for RCT1 and RCT2, respectively.

Supplementary Table 6 The demographic and dietary intake after intervention in the two RCTs

|  | RCT1 | | | | RCT2 | | |
| --- | --- | --- | --- | --- | --- | --- | --- |
|  | Control  (N=40) | 100g butter per day  (N=37) | 120g butter per day  (N=37) | *P* -value | Control  (N=52) | HF-diet  (N=58) | *P*-value |
| Energy (kcal/d) | 2147 (94) | 2905(178) | 3145(146) | <0.001 | 2251 (96) | 2565 (23.3) | <0.001 |
| Protein (g/d) | 74.3 (2.7) | 74.3 (3.2) | 74.1 (3.3) | 0.984 | 79.1 (7.7) | 90.6 (5.1) | <0.001 |
| Carbohydrate (g/d) | 323.8 (9.7) | 326.8(9.0) | 328.1(8.2) | 0.888 | 334.1 (4.2) | 336.1(12.1) | 0.223 |
| Total Fat (g/d) | 58.4 (4.3) | 167.8 (3.4) | 190.1 (1.9) | <0.001 | 50.7 (4.3) | 105.8 (0.8) | <0.001 |
| SFA (g/d) | 14.1 (1.9) | 96.4 (3.0) | 112.9 (2.7) | <0.001 | 17.7 (3.4) | 44.7 (1.5) | <0.001 |
| C14:0 (g/d) | 0.3 (0.1) | 14.1 (0.5) | 17.0 (0.6) | <0.001 | 0.6 (0.1) | 1.8 (0.3) | <0.001 |
| C15:0 (g/d) | 0.1 (0.1) | 1.7 (0.1) | 2.1 (0.1) | <0.001 | 0.03 (0.01) | 0.07 (0.01) | <0.001 |
| C16:0(g/d) | 10.5 (1.6) | 49.2 (2.6) | 54.9 (1.9) | <0.001 | 11.8 (1.6) | 31.6 (4.7) | <0.001 |
| C17:0 (g/d) | 0.1 (0.1) | 0.9 (0.1) | 1.1 (0.1) | <0.001 | 0.1 (0.1) | 0.2 (0.1) | <0.001 |
| C18:0 (g/d) | 3.1 (0.5) | 22.0 (0.9) | 26.0 (1.9) | <0.001 | 4.7 (0.8) | 12.9 (1.7) | <0.001 |
| C19:0 (g/d) | 0.15 (0.02) | 0.20 (0.02) | 0.20 (0.05) | <0.001 | 0.05 (0.01) | 0.14 (0.02) | <0.001 |
| C20:0 (g/d) | 0.12 (0.02) | 0.31 (0.02) | 0.33 (0.03) | <0.001 | 0.18 (0.03) | 0.50 (0.10) | <0.001 |
| C22:0 (g/d) | 0.05 (0.01) | 0.07 (0.01) | 0.07 (0.01) | <0.001 | 0.02 (0.01) | 0.05 (0.01) | <0.001 |
| MUFA (g/d) | 24.9 (3.3) | 47.0 (4.0) | 51.7 (5.5) | <0.001 | 22.7 (3.2) | 42.9 (6.3) | <0.001 |
| PUFA (g/d) | 13.4 (1.8) | 19.3 (1.0) | 20.5 (1.8) | <0.001 | 13.6 (2.4) | 17.7 (2.1) | <0.001 |

Continuous variables was presented as mean and SD; Categorical variables was presented as numbers and percentage; SFA, saturated fatty acid; MUFA, monounsaturated fatty acids; PUFA, polyunsaturated fatty acids;

One-way ANOVA and t-test was used to probe the differences across groups for RCT1 and RCT2, respectively.

Supplementary Table-7 Baseline serum profiles of fatty acids and amino acids in the two RCTs

|  | RCT1 | | | | RCT2 | | |
| --- | --- | --- | --- | --- | --- | --- | --- |
| Fatty acids | Control  (N=40) | 100g butter  Per day  (N=37) | 120g butter  Per day  (N=37) | *P*-value | Control  (N=52) | HF-diet  (N=58) | *P*-value |
| C14:0 (μg/ml) | 2.23(0.75) | 2.06(0.52) | 2.28(0.81) | 0.392 | 2.09(0.87) | 2.07(0.54) | 0.892 |
| C16:0 (μg/ml) | 125.5(27.1) | 122.8(33.5) | 131.1(40.8) | 0.590 | 114.9(28.3) | 123.2(34.6) | 0.175 |
| C16:1 (μg/ml) | 3.78(1.90) | 3.51(1.36) | 3.63(1.14) | 0.749 | 3.57(1.09) | 3.44(1.45) | 0.586 |
| C18:0 (μg/ml) | 60.2(18.5) | 57.1(12.9) | 58.6(17.0) | 0.727 | 51.7(11.3) | 54.0(7.6) | 0.196 |
| C18:1 (μg/ml) | 73.4(12.9) | 76.3(17.0) | 72.6(13.5) | 0.542 | 71.7(16.0) | 71.4(19.5) | 0.933 |
| C18:2 (μg/ml) | 81.4(63.0) | 71.1(32.7) | 77.8(52.2) | 0.700 | 95.2(15.4) | 90.4(42.4) | 0.440 |
| C18:3-α (μg/ml) | 8.39(1.53) | 8.28(1.03) | 8.17(0.81) | 0.733 | 8.04(2.85) | 7.69(1.18) | 0.398 |
| C18:3-γ (μg/ml) | 1.69(1.88) | 1.52(0.79) | 1.82(1.58) | 0.795 | 1.13(0.48) | 1.23(0.84) | 0.446 |
| C18:4 (μg/ml) | 0.71(0.48) | 0.75(0.40) | 0.81(0.43) | 0.771 | 0.71(0.62) | 0.68(0.48) | 0.746 |
| C20:2 (μg/ml) | 0.16(0.15) | 0.14(0.10) | 0.15(0.11) | 0.783 | 0.15(0.05) | 0.16(0.13) | 0.598 |
| C20:3 (μg/ml) | 8.19(2.38) | 7.69(1.95) | 7.72(1.86) | 0.530 | 8.21(1.46) | 7.99(1.46) | 0.441 |
| C20:4 (μg/ml) | 46.0(6.3) | 44.8(5.0) | 45.5(5.5) | 0.662 | 49.3(10.2) | 47.4(9.5) | 0.317 |
| C20:5 (μg/ml) | 22.1(6.6) | 21.8(11.6) | 22.0(10.8) | 0.995 | 22.7(10.0) | 21.0(9.2) | 0.355 |
| C22:4 (μg/ml) | 0.86(0.72) | 0.82(0.79) | 0.61(0.54) | 0.287 | 0.88(0.40) | 0.85(0.62) | 0.779 |
| C22:5 (μg/ml) | 1.36(0.33) | 1.27(0.33) | 1.26(0.40) | 0.456 | 1.28(0.37) | 1.23(0.32) | 0.462 |
| C22:6 (μg/ml) | 9.29(2.25) | 9.43(2.59) | 8.55(2.07) | 0.245 | 9.41(4.60) | 9.06(2.84) | 0.623 |
| Amino acids |  |  |  |  |  |  |  |
| γ-Aminobutyric acid (umol/L) | 505.6(190.3) | 499.7(203.1) | 523.5(193.9) | 0.864 | 467.3(72.6) | 474.2(61.0) | 0.591 |
| Valine (umol/L) | 34.2(5.1) | 34.5 (5.3) | 35.5(5.0) | 0.475 | 37.9(5.2) | 38.4(4.4) | 0.554 |
| L-Leucine (umol/L) | 6.67(0.99) | 6.80(1.27) | 7.16(1.24) | 0.181 | 6.47(1.48) | 6.86(1.06) | 0.116 |
| L-Isoleucine (umol/L) | 7.52(1.36) | 7.39(1.74) | 7.93(1.64) | 0.309 | 7.25(1.63) | 7.60(1.31) | 0.223 |
| Tyrosine (umol/L) | 5.67(1.31) | 5.45(1.57) | 5.67(1.66) | 0.766 | 5.41(1.12) | 5.57(1.37) | 0.502 |
| L-Threonine (umol/L) | 46.0 (9.1) | 48.0(10.1) | 48.0(8.9) | 0.565 | 51.2(9.9) | 48.3(7.1) | 0.081 |
| Taurine (umol/L) | 188.7(98.1) | 192.3(101.6) | 205.8(117.4) | 0.764 | 175.1(12.6) | 173.4(10.1) | 0.432 |
| L-Serine (umol/L) | 58.9(8.3) | 59.9(9.7) | 61.6(8.9) | 0.441 | 61.7(7.7) | 59.6(7.2) | 0.149 |
| L-Proline (umol/L) | 168.7(47.8) | 189.6(53.2) | 178.5(53.2) | 0.218 | 173.7(64.3) | 165.1(43.2) | 0.404 |
| L-Phrnylalanine (umol/L) | 18.2(4.4) | 18.8(4.7) | 19.8(5.0) | 0.357 | 20.4 (2.0) | 20.1(2.6) | 0.460 |
| L-Ornithine (umol/L) | 26.5 (7.7) | 27.4 (7.3) | 29.7 (9.2) | 0.204 | 29.2(6.8) | 27.3(5.1) | 0.101 |
| L-Methionine (umol/L) | 3.32 (0.78) | 3.34(0.83) | 3.28 (0.72) | 0.936 | 3.21(0.54) | 3.15(0.48) | 0.532 |
| L-lysine (umol/L) | 52.7 (11.1) | 52.4 (12.6) | 57.0 (14.8) | 0.241 | 50.5 (6.5) | 51.0(6.9) | 0.680 |
| L-histidine (umol/L) | 230.4(53.3) | 232.1(54.4) | 235.4(58.0) | 0.922 | 217.5(24.5) | 215.0(24.5) | 0.606 |
| Glycine (umol/L) | 169.6(80.9) | 187.9(61.0) | 180.4(52.9) | 0.480 | 192.3(33.5) | 184.1(24.2) | 0.153 |
| L-Glutamic Acid (umol/L) | 27.3(7.9) | 26.7(8.3) | 28.2(8.4) | 0.725 | 29.3(6.4) | 27.3(5.5) | 0.073 |
| L-Citrulline (umol/L) | 2.72(0.56) | 2.74(0.69) | 2.83(0.59) | 0.683 | 2.65(0.68) | 2.72(0.45) | 0.497 |
| L-Asparagine (umol/L) | 16.3(3.1) | 17.0(3.6) | 17.5(3.3) | 0.290 | 15.6(2.25) | 15.3(2.68) | 0.511 |
| L-Arginine (umol/L) | 28.9(6.4) | 29.1(6.5) | 31.4(8.0) | 0.232 | 35.7(5.4) | 37.1(6.0) | 0.208 |
| L-Alanine (umol/L) | 129.7(28.2) | 130.8(26.3) | 136.0(34.6) | 0.619 | 122.0(22.4) | 118.2(22.4) | 0.399 |
| L-Aminobutyric Acid (umol/L) | 1.66(0.57) | 1.87(0.93) | 1.96(0.99) | 0.284 | 1.87(0.79) | 1.93(0.49) | 0.663 |

Supplementary Table-8 serum profiles of fatty acids and amino acids after the intervention in the two RCTs

|  | RCT1 | | | | RCT2 | | |
| --- | --- | --- | --- | --- | --- | --- | --- |
| Fatty acids | Control  (N=40) | 100g butter  Per day  (N=37) | 120g butter  Per day  (N=37) | *P*-value | Control  (N=52) | HF-diet  (N=58) | *P*-value |
| C14:0 (μg/ml) | 2.21 (0.62) | 3.67 (1.17) | 4.08 (1.03) | <0.001 | 2.18 (1.31) | 3.01 (1.61) | 0.004 |
| C16:0 (μg/ml) | 119.5 (22.1) | 123.4 (29.2) | 137.5 (32.4) | 0.024 | 112.0 (26.1) | 106.1 (25.8) | 0.239 |
| C16:1 (μg/ml) | 3.58 (0.64) | 3.90 (1.47) | 4.23 (0.99) | 0.052 | 3.36 (1.10) | 3.43 (1.37) | 0.767 |
| C18:0 (μg/ml) | 54.3 (14.1) | 56.7 (17.5) | 69.4 (21.5) | 0.001 | 53.1 (12.6) | 52.4 (11.6) | 0.755 |
| C18:1 (μg/ml) | 70.3 (13.3) | 64.0 (13.5) | 62.9 (12.3) | 0.053 | 66.7 (20.7) | 71.5 (18.9) | 0.215 |
| C18:2 (μg/ml) | 77.4 (38.4) | 65.5(48.3) | 62.2 (52.2) | 0.019 | 85.3 (24.3) | 94.2 (16.8) | 0.030 |
| C18:3-α (μg/ml) | 8.15 (0.83) | 7.95 (0.77) | 7.85 (0.74) | 0.254 | 6.67 (2.52) | 7.83 (3.01) | 0.028 |
| C18:3-γ (μg/ml) | 1.57 (1.07) | 2.36 (1.81) | 2.10 (2.71) | 0.458 | 1.31 (1.22) | 1.20 (0.58) | 0.590 |
| C18:4 (μg/ml) | 0.76 (0.24) | 0.84 (0.36) | 0.91 (0.39) | 0.478 | 0.62 (0.84) | 0.68 (0.67) | 0.698 |
| C20:2 (μg/ml) | 0.16 (0.17) | 0.17 (0.19) | 0.17 (0.14) | 0.911 | 0.16 (0.11) | 0.14 (0.06) | 0.527 |
| C20:3 (μg/ml) | 8.04 (1.95) | 6.82 (1.97) | 6.09 (1.27) | <0.001 | 8.16 (3.01) | 8.60 (3.09) | 0.453 |
| C20:4 (μg/ml) | 45.7 (5.7) | 42.7 (4.4) | 42.0 (5.2) | <0.001 | 42.1 (12.4) | 48.9 (10.9) | 0.003 |
| C20:5 (μg/ml) | 20.4 (8.7) | 17.7 (7.5) | 24.7 (12.7) | 0.026 | 23.9 (9.1) | 24.4 (9.7) | 0.762 |
| C22:4 (μg/ml) | 0.87 (0.90) | 0.83 (0.84) | 0.91 (1.02) | 0.937 | 0.74 (0.41) | 1.01 (0.99) | 0.60 |
| C22:5 (μg/ml) | 1.28 (0.27) | 1.11 (0.43) | 1.06 (0.39) | <0.001 | 1.24 (0.36) | 1.21 (0.37) | 0.660 |
| C22:6 (μg/ml) | 9.30 (2.02) | 8.40 (2.08) | 7.23 (1.88) | <0.001 | 8.58 (4.95) | 9.49 (7.77) | 0.459 |
| Amino acids |  |  |  |  |  |  |  |
| γ-Aminobutyric acid (umol/L) | 481.8 (130.8) | 545.1 (109.1) | 562.9 (136.0) | 0.027 | 447.2 (48.7) | 447.9 (68.1) | 0.950 |
| Valine (umol/L) | 35.3 (13.7) | 33.2 (5.5) | 33.8 (7.9) | 0.657 | 35.9 (5.7) | 35.4 (4.6) | 0.589 |
| L-Leucine (umol/L) | 6.56 (7.27) | 8.02 (9.27) | 11.0 (12.2) | 0.166 | 6.44 (2.27) | 6.72 (1.35) | 0.435 |
| L-Isoleucine (umol/L) | 9.91 (9.46) | 8.67 (3.89) | 10.6 (5.79) | 0.508 | 7.60 (1.67) | 7.26 (2.93) | 0.448 |
| Tyrosine (umol/L) | 6.27 (1.88) | 8.51 (3.70) | 9.49 (6.98) | 0.009 | 5.31 (1.03) | 6.00 (1.41) | 0.005 |
| L-Threonine (umol/L) | 47.8 (15.3) | 50.8 (17.5) | 49.4 (15.9) | 0.768 | 45.7 (8.0) | 47.6 (9.1) | 0.246 |
| Taurine (umol/L) | 177.2 (153.8) | 178.7 (153.7) | 217.1 (177.3) | 0.546 | 181.4 (8.8) | 185.5 (15.1) | 0.093 |
| L-Serine (umol/L) | 60.6 (24.0) | 64.7 (25.7) | 64.5 (22.3) | 0.741 | 62.2 (7.1) | 59.2 (9.3) | 0.065 |
| L-Proline (umol/L) | 174.9 (83.3) | 203.2 (105.8) | 182.6 (115.0) | 0.486 | 183.1 (56.0) | 187.0 (48.5) | 0.695 |
| L-Phrnylalanine (umol/L) | 19.5 (10.4) | 18.3 (6.9) | 19.5 (7.0) | 0.769 | 21.7 (2.7) | 22.8 (3.6) | 0.081 |
| L-Ornithine (umol/L) | 28.3 (7.3) | 33.3 (8.8) | 34.7 (11.1) | 0.011 | 28.6 (6.4) | 28.1 (7.9) | 0.875 |
| L-Methionine (umol/L) | 3.58 (1.37) | 3.94 (1.97) | 4.63 (3.80) | 0.274 | 3.47 (0.60) | 3.50 (0.68) | 0.773 |
| L-lysine (umol/L) | 54.0 (17.8) | 54.8 (16.1) | 55.9 (17.0) | 0.903 | 51.4 (6.0) | 53.1 (8.0) | 0.219 |
| L-histidine (umol/L) | 217.9 (57.3) | 187.5 (74.0) | 185.0 (68.5) | 0.047 | 204.5 (35.6) | 223.6 (26.0) | 0.002 |
| Glycine (umol/L) | 179.6 (80.9) | 162.8 (65.0) | 147.6 (60.8) | 0.033 | 163.3 (27.8) | 196.0 (41.0) | 0.001 |
| L-Glutamic Acid (umol/L) | 23.6 (5.9) | 24.9 (7.8) | 25.2 (7.5) | 0.654 | 27.1 (6.3) | 27.7 (6.2) | 0.622 |
| L-Citrulline (umol/L) | 2.78 (0.87) | 3.32 (0.73) | 3.54 (1.56) | 0.017 | 2.40 (0.53) | 2.74 (0.64) | 0.003 |
| L-Asparagine (umol/L) | 15.0 (3.8) | 16.3 (6.7) | 15.2 (4.0) | 0.525 | 14.8 (2.33) | 15.1 (2.18) | 0.476 |
| L-Arginine (umol/L) | 35.5 (7.5) | 37.6 (7.8) | 37.7 (8.6) | 0.454 | 37.7 (8.00) | 38.8 (7.82) | 0.460 |
| L-Alanine (umol/L) | 139.6 (41.5) | 171.1 (49.4) | 181.3 (56.0) | 0.001 | 110.4 (22.3) | 115.4 (24.0) | 0.260 |
| L-Aminobutyric Acid (umol/L) | 1.79 (0.48) | 2.49 (1.45) | 2.93 (1.47) | <0.001 | 2.03 (0.54) | 1.97 (0.86) | 0.650 |

Supplementary Table-9 Baseline characteristic of studying variables in the prospective cohort by status of T2DM

|  | Without diabetes  (N=3692) | New cases of type 2 diabetes  (N=365) | *P*-value |
| --- | --- | --- | --- |
| Age (years) | 49.3 (9.6) | 52.4 (9.3) | <0.001 |
| Men [n (%)] | 1203 (32.6) | 149 (40.8) | 0.002 |
| BMI (kg/m2) | 24.7 (3.4) | 26.2 (3.5) | <0.001 |
| WC (cm) | 84.8 (10.0) | 89.0 (9.5) | <0.001 |
| Weight gain during follow-up (kg) | 0.65 (6.19) | 1.36 (8.53) | <0.001 |
| Smoking [n (%)] | 569 (15.4) | 67 (18.4) | 0.325 |
| Drinking [n (%)] | 1310 (35.5) | 116 (31.8) | 0.168 |
| Over high school [n (%)] | 2683 (72.7) | 238 (65.2) | 0.002 |
| Light physical activity [n (%)] | 3062 (82.9) | 287 (78.6) | 0.091 |
| Regular exercise habitus [n (%)] | 1699 (46.0) | 167 (45.8) | 0.923 |
| Family history of diabetes [n (%)] | 533 (14.4) | 69 (18.9) | 0.022 |
| Hypertension [n (%)] | 1270 (34.5) | 189 (51.8) | <0.001 |
| Energy intake (kcal/d) | 2365 (872) | 2438 (912) | 0.127 |
| SFA (g/d) | 15.9 (7.4) | 17.0 (8.6) | 0.042 |
| TC (mmol/L) | 5.11 (1.00) | 5.24 (1.00) | 0.021 |
| TG (mmol/L) | 1.65 (1.57) | 2.21 (1.93) | <0.001 |
| HDL-C (mmol/L) | 1.27 (0.32) | 1.17 (0.29) | <0.001 |
| FS-glucose (mmol/L) | 4.46 (0.65) | 5.06 (0.87) | <0.001 |
| 2h-glucose (mmol/L) | 5.70 (1.54) | 7.01 (1.87) | <0.001 |
| HOMA-IR | 1.00 (0.63) | 1.24 (0.63) | <0.001 |
| Leucine (umol/L) | 15.2 (7.6) | 15.8 (7.1) | 0.126 |
| Isoleucine (umol/L) | 23.0 (10.3) | 24.8 (10.6) | 0.001 |
| Valine (umol/L) | 97.4 (25.6) | 103.7 (29.1) | <0.001 |
| C14:0(μg/ml) | 2.68 (1.78) | 3.48 (2.12) | <0.001 |
| C20:4(μg/ml) | 44.3 (14.1) | 44.0 (15.1) | 0.633 |
| Tyrosine (umol/L) | 21.8 (11.8) | 23.7 (12.7) | 0.019 |
| Histidine (umol/L) | 164.5 (15.7) | 162.0 (24.4) | 0.505 |
| Glycine (umol/L) | 193.3 (100.1) | 192.4 (90.8) | 0.825 |
| MA-TGH | 3.21 (3.23) | 4.31 (3.31) | <0.001 |

Continuous variables are presented mean and Standard Deviation; categorical variables are presented as numbers and percentage.

Supplementary Table 10 HRs and 95%CI across quartiles of the biomarker in the men and women

|  | Quartile 1 | Quartile 2 | Quartile 3 | Quartile 4 | *P* _for trend_ |
| --- | --- | --- | --- | --- | --- |
| Men |  |  |  |  |  |
| Case/N | 20/338 | 31/338 | 38/338 | 60/338 |  |
| Model1 | 1 (Ref.) | 1.79 (0.99-3.23) | 2.36 (1.34-4.14) | 4.13 (2.41-7.09) | <0.001 |
| Model2 | 1 (Ref.) | 1.70 (0.94-3.10) | 1.97 (1.10-3.51) | 3.16 (1.75-5.72) | <0.001 |
| Model3 | 1 (Ref.) | 1.74 (0.95-3.18) | 2.01 (1.12-3.60) | 3.18 (1.74-5.82) | <0.001 |
| Women |  |  |  |  |  |
| Case/N | 30/677 | 38/676 | 57/675 | 91/677 |  |
| Model1 | 1 (Ref.) | 1.03 (0.62-1.70) | 2.07 (1.33-3.24) | 2.58 (1.68-3.94) | <0.001 |
| Model2 | 1 (Ref.) | 1.06 (0.64-1.75) | 1.78 (1.14-2.79) | 2.00 (1.29-3.11) | <0.001 |
| Model3 | 1 (Ref.) | 1.04 (0.62-1.74) | 1.74 (1.09-2.78) | 1.93 (1.18-3.14) | 0.001 |

In the Mode1, age, sex, BMI, WC, weight gain, smoke use, alcohol use, education level, family history of diabetes, energy intake, regular exercise habitus, physical activity levels, and hypertension were included;

Model 2 additionally adjusted for TG, HDL-C, TC, FS-glucose, 2h-glucose, and HOMA-IR based on Model 1.

Model 3 additionally adjusted for serum leucine, isoleucine, and valine based on Model 2.

BMI, body mass index; WC, waist circumference; TG, triglycerides; HDL-C, high density lipoprotein cholesterol; TC, total cholesterol; FS, fasting serum;


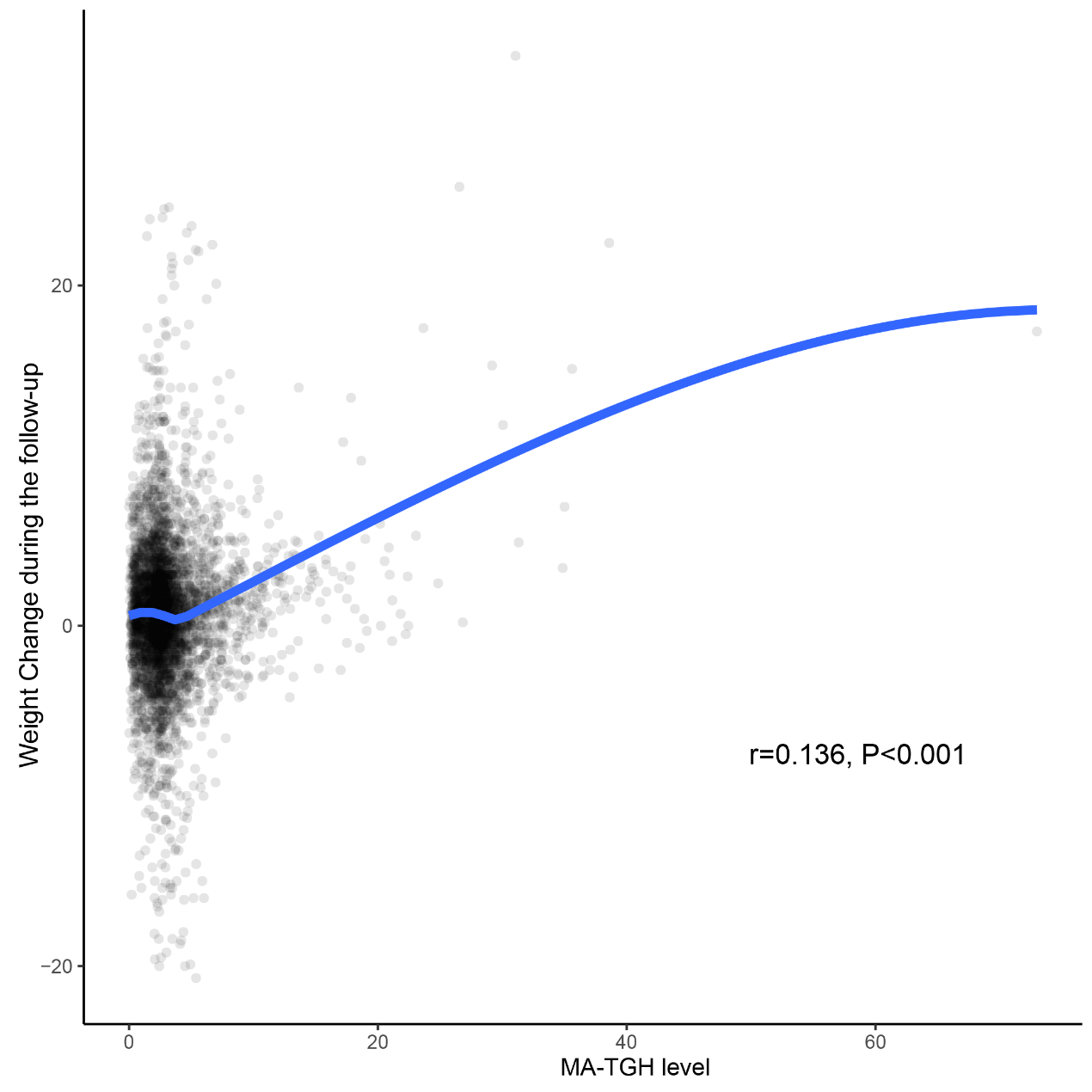


**Supplementary Figure-1 scatter plot between weight change and MA-TGH level**
